# The Impacts of Fish Oil and/or Probiotic Intervention on Low-Grade Inflammation, IGFBP-1 and MMP-8 in Pregnancy: A Randomized, Placebo-Controlled, Double-Blind Clinical Trial

**DOI:** 10.3390/biom11010005

**Published:** 2020-12-22

**Authors:** Noora Houttu, Kati Mokkala, Ella Koivuniemi, Outi Pellonperä, Juuso Juhila, Timo Sorsa, Kirsi Laitinen

**Affiliations:** 1 Institute of Biomedicine, Research Centre for Integrative Physiology and Pharmacology, University of Turku, 20520 Turku, Finland; kamamo@utu.fi (K.M.); elmkoi@utu.fi (E.K.); kirsi.laitinen@utu.fi (K.L.); 2 Department of Obstetrics and Gynecology, University of Turku and Turku University Hospital, 20520 Turku, Finland; ouirpe@utu.fi; 3 Actim Oy, 02180 Espoo, Finland; Juuso.Juhila@actimtest.com; 4 Department of Oral and Maxillofacial Disease, University of Helsinki and Helsinki University Hospital, 00014 Helsinki, Finland; timo.sorsa@helsinki.fi; 5 Department of Oral Diseases, Karolinska Institutet, 141 04 Huddinge, Sweden

**Keywords:** low-grade inflammation, IGFBP-1, MMP-8, obesity, pregnancy

## Abstract

Background: We investigated the impact of fish oil and/or probiotics on serum and vaginal inflammatory and metabolic proteins and their relation to the onset of gestational diabetes mellitus (GDM). Methods: Overweight/obese pregnant women received fish oil + placebo, probiotics + placebo, fish oil + probiotics or placebo + placebo from early pregnancy until six months postpartum (fish oil: 1.9 g docosahexaenoic acid and 0.22 g eicosapentaenoic acid; probiotics: *Lactobacillus rhamnosus* HN001 and *Bifidobacterium animalis* ssp. *lactis* 420, 10^10^ colony-forming units each). Serum high sensitivity C-reactive protein (hsCRP) and serum/vaginal (s/v) phosphorylated insulin-like growth factor binding-protein-1 (phIGFBP-1), IGFBP-1 and matrix metalloproteinase 8 (MMP-8) were analyzed. GDM was diagnosed according to 2 h 75 g OGTT. Results: The intervention had no impact on the change in proteins during pregnancy. Nevertheless, s-MMP-8 decreased and s-IGFBP-1 increased more in obese than in overweight women in the fish oil + probiotics group, while a decrease in s-MMP-8 was seen in obese women and an increase was seen in overweight women in the probiotics + placebo group. The late pregnancy s-phIGFBP-1 was higher in women who developed GDM in fish oil + probiotics-group compared to fish oil + placebo-group. The concentrations of s-phIGFBP-1 (635.9 ± 315.3 ng/mL vs. 753.2 ± 335.1 ng/mL, *p* = 0.005) and s-IGFBP-1 (3.78 ± 0.72 ng/mL vs. 3.96 ± 0.69 ng/mL, *p* = 0.042) were lower in early pregnancy in women who developed GDM than in women remaining healthy. Conclusions: The intervention per se had no impact on the proteins, but obesity and GDM may modify the effect. IGFBPs may affect the development of GDM.

## 1. Introduction

Both increased low-grade inflammation and higher adiposity have been linked to gestational diabetes mellitus (GDM) [1], which is a serious condition in pregnancy, increasing the risk of metabolic complications in both mother and child. Mechanistically, higher adiposity increases the concentration of circulating pro-inflammatory markers which may lead to insulin resistance, simultaneously decreasing the levels of insulin-like growth factor binding-protein 1 (IGFBP-1) and increasing the bioavailability of IGF-1 [2]. Pregnancy itself causes changes in the metabolism, specifically in insulin and glucose metabolism, but may result in excessive disturbances and thus GDM. IGFBP-1, and especially the phosphorylated (ph) form of IGFBP-1, and matrix metalloproteinase 8 (MMP-8) measured either from vaginal or cervical fluid have been used as markers for preterm birth [3,4] and intra-amniotic inflammation [5,6], respectively. Moreover, there is preliminary evidence that lower serum concentrations IGFBP-1 and higher serum concentrations of MMP-8 may be linked to low-grade inflammation [7,8] and obesity [9,10], providing initial evidence of use as metabolic markers as well.

The risk of GDM could be potentially modulated by reducing low-grade inflammation and restoring insulin metabolism via the IGF and MMP systems. Based on previous studies, n-3 long-chain polyunsaturated fatty acids (LC-PUFA) and probiotics have displayed promise in regulating the levels of low-grade inflammatory markers, such as high-sensitivity C-reactive protein (hsCRP), and metabolic markers, IGFBPs and MMPs. The level of hsCRP has been shown to decline after interventions with a probiotic [11,12] or a n-3 LC-PUFA [13,14,15,16] in pregnant and non-pregnant subjects although not all reports agree with these findings [12,17,18]. In terms of IGFBP-1 and MMP-8, dietary intervention studies in pregnant women are lacking. However, in non-pregnant adult subjects, the consumption of n-3 LC-PUFA has been shown to increase the serum concentration of IGF-1 and IGFBP-3 [19,20,21], whereas probiotic administration has been demonstrated to increase hepatic IGFBP-2 [22] and white adipose tissue IGFBP-3 [23] concentrations in experimental animals. With respect to MMP, probiotic consumption has been shown to increase MMP-9 levels in saliva [24] and decrease those of MMP-8 in gingival crevicular fluid [25]. Therefore, we hypothesized that it may be possible to interfere with obesity-related excessive inflammation and disturbed metabolism by administering n-3 LC-PUFA (fish oil) and/or probiotics to overweight and obese pregnant women, a risk group for developing pregnancy-related complications.

The objectives are firstly to investigate the impact of fish oil and/or probiotic supplementation from early pregnancy onwards on the serum hsCRP concentration and serum and vaginal levels of IGFBP-1, phIGFBP-1, and MMP-8 and secondly to determine whether these low-grade inflammatory and metabolic markers could predict the diagnosis of GDM. As there are not many studies on phIGFBP-1, IGFBP-1 and MMP-8 during pregnancy, in this longitudinal study, we have also determined the changes in serum and vaginal levels of phIGFBP-1, IGFBP-1 and MMP-8 as well as that of hsCRP from early to late pregnancy.

## 2. Materials and Methods

### 2.1. Study Design and Subjects

**Figure 1:**
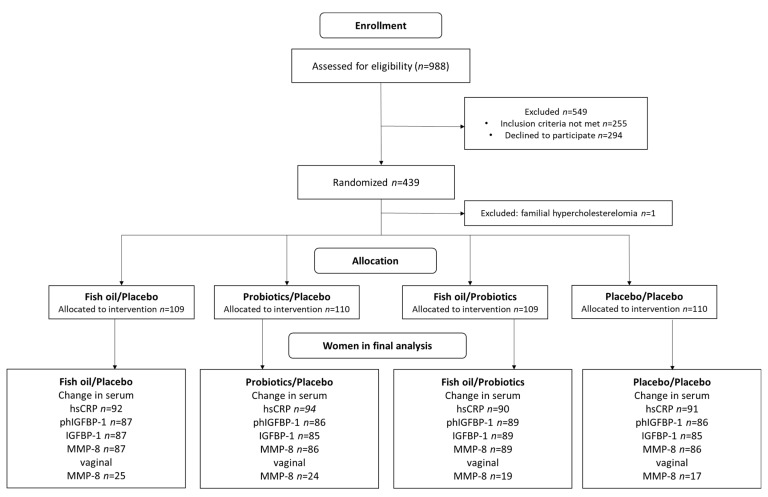
Flow chart. hsCRP, high-sensitivity C-reactive protein; phIGFBP-1, phosphorylated insulin-like growth factor binding-protein 1; MMP-8, matrix metalloproteinase 8.

**Figure 2:**
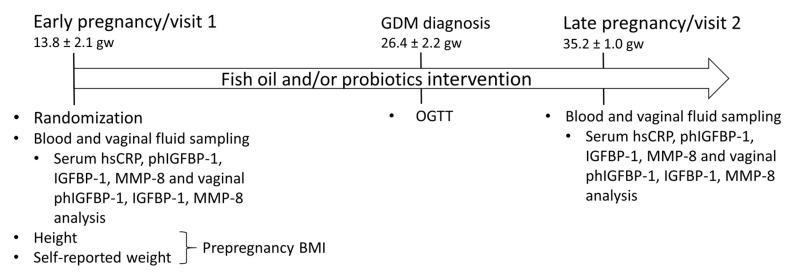
Timeline of the study. gw, gestational weeks; hsCRP, high-sensitivity C-reactive protein; phIGFBP-1, phosphorylated insulin-like growth factor binding-protein 1; MMP-8, matrix metalloproteinase 8; BMI, body mass index; OGTT, oral glucose tolerance test.

The design of this study has been described in detail previously [26]. In this double-blind, placebo-controlled randomized trial, the impact of fish oil and/or probiotics on maternal and child health are studied (ClinicalTrials.gov, NCT01922791). The trial was conducted in the Turku University Hospital and University of Turku in Finland. The recruitment started in October 2013 and was completed in July 2017. This study complied with the Declaration of Helsinki as revised in 2000. The Ethics Committee of the Hospital District of South-West Finland (115/180/2012) approved the study protocol. Informed consent form was provided by all participants. A total of 439 overweight and obese women that are hence at an increased risk for GDM were recruited to the clinical trial and were randomized into four intervention groups (fish oil + placebo, probiotics + placebo, fish oil + probiotics and placebo + placebo). One woman who was later discovered to have familiar hypercholesterolemia was excluded. The study flow is presented in Figure 1 and the study timeline in Figure 2.

Women consumed two fish oil capsules and one probiotic capsule daily from early pregnancy/first study visit until 6 months postpartum. The fish oil capsules (Croda Europe Ltd., Leek, UK, Incromega E1070) contained 2.4 g of n-3 fatty acids; 1.9 g docosahexaenoic acid (22:6 n-3, DHA), 0.22 g eicosapentaenoic acid (20:5 n-3, EPA) and the remaining amount other n-3 fatty acids. The placebo capsules for fish oil consisted of 2.4 g medium-chain fatty acids (capric acid C8 54.6% and caprylic acid C10 40.3%) and the size, shape, color and lemon flavor were the same as in fish oil capsules. Probiotic capsules contained *Lactobacillus rhamnosus* HN001 (ATCC SD5675; DuPont, Niebüll, Germany) and *Bifidobacterium animalis* ssp. *lactis* 420 (DSM 22089; DuPont), each with 10^10^ colony-forming units per capsule. The placebo for the probiotics consisted of microcrystalline cellulose and the size, shape and color were same as the probiotic capsules. The intervention supplements for the on-going trial (ClinicalTrials.gov, NCT01922791), which aims to investigate both mother and child health outcomes, were selected based on the previous scientific knowledge. The probiotic *L. rhamnosus* HN001 is a well characterized probiotic [27] and *B. lactis* 420 is a novel probiotic with demonstrated health benefits related to metabolism in an animal study [28] and inflammation in humans [29,30]. LC-PUFA, in this case fish oil, which is rich in DHA and EPA, are known inflammation-resolving dietary factors [31] and are important for fetal and child development [32], and may possibly reduce insulin resistance [33].

Here, we studied secondary outcomes of the trial, the low-grade inflammation and IGFBP-1 and MMP-8 as determined from serum and vagina. The participants in whom a serum or vaginal sample was available at early or late pregnancy, resulting in 434 pregnant women, were included in the analyses. The clinical characteristics of the study population included in this report were essentially the same as described in detail in Pellonperä et al. [26]. Women attended two study visits during gestation at early (mean 13.8 ± 2.1 gestational weeks) and late (mean of 35.2 ± 1.0 gestational weeks) pregnancy.

Height was measured by a wall stadiometer in 0.1 cm accuracy. Pre-pregnancy body mass index (BMI) (kg/m^2^) was calculated by dividing self-reported weight in kilograms, obtained from welfare women clinic records, by height measured in the early pregnancy. Overweight was defined as BMI ≥25 kg/m^2^, while obesity as BMI ≥30 kg/m^2^. GDM was diagnosed with a 2 h 75 g oral glucose tolerance test (OGTT) if one or more values were at or above the threshold levels: 0 h ≥5.3, 1 h ≥10.0, 2 h ≥8.6 mmol/L, according to the Finnish Current Care guidelines [34], at a mean of 26.4 ± 2.2 weeks of gestation. OGTT was offered also to high-risk women (BMI ≥ 35 kg/m^2^, previous GDM, glucosuria, polycystic ovarian syndrome, or family risk of diabetes) at early pregnancy, 12–16 weeks of gestation, and the women who were diagnosed with GDM at early pregnancy were excluded from the analysis at the onset of GDM.

### 2.2. Sampling and Analyses

After at least 9 h of overnight fasting, a blood sample was drawn from the antecubital vein of the mothers. The serum was separated and analyzed for hsCRP and the rest of the samples were kept at −80 °C until analyzed for phIGFBP-1, IGFBP-1 and MMP-8 (Medix Biochemica, Espoo, Finland). HsCRP i.e., low-grade inflammation, was analyzed by using an automated colorimetric immunoassay on the Dade Behring Dimension RXL autoanalyzer (Siemens Healthcare, Camberly, Surrey, UK) in a certified laboratory (TYKSLAB, the Hospital District of Southwest Finland); the lower limit of detection was 0.1 mg/L. The data are expressed as mg/L.

Vaginal samples were obtained by research coordinator using sterile swabs (Puritan Sterile Polyester swabs, Puritan Medical Products Company Co. LLC, Guilford, CT, USA) and were dissolved in PROM/Partus Specimen Extraction (Medix Biochemica, Espoo, Finland) and MMP-8 buffer (Medix Biochemica, Espoo, Finland) solutions. The solutions were kept at −20 °C until further analysis.

Concentrations of serum and vaginal IGFBP-1 and phIGFBP-1 were measured by two immunoenzymometric assays using monoclonal antibodies (Medix Biochemica, Espoo, Finland). The IGFBP-1 assay employing monoclonal antibody 6305 detects the non-phosphorylated and the less phosphorylated isoforms of IGFBP-1, whereas the phIGFBP-1 assay with monoclonal antibody 6303 recognizes the highly phosphorylated forms [35]. The detection limit of both assays was 0.3 ng/mL [36]. The data are expressed as ng/mL.

MMP-8 was quantified with a solid-phase immunoenzymometric assay (MMP-8 IEMA, Medix Biochemica, Espoo, Finland) [5,6]. This sandwich assay uses two monoclonal antibodies against human MMP-8. Microplate wells are coated with one monoclonal antibody against MMP-8. The other antibody is conjugated to HRP forming the enzyme conjugate used to detect the presence of MMP-8. Analyses were performed according to the manufacturer’s instructions, and the absorbance of the solutions in the wells was measured at 414 nm using a microplate reader (Multiskan, Thermo Fisher Scientific, Vantaa, Finland). The detection limit was 0.04 ng/mL [37]. The data are expressed as ng/mL.

### 2.3. Statistics

The power calculations of this trial were originally calculated to evaluate the effects of fish oil and/or probiotic intervention on glycemic status and GDM prevention [26]. The predefined secondary outcomes were serum hsCRP and serum and vaginal phIGFBP-1, IGFBP-1 and MMP-8 in this secondary analysis of the main trial. Based on previous studies investigating the effect of fish oil or probiotics on hsCRP during pregnancy [11,12,13,14,15,16], the number of pregnant women in our study was estimated to be adequate. The effect of fish oil or probiotics on phIGFBP-1, IGFBP-1 and MMP-8 during pregnancy has not been studied previously. Visual inspection of histograms and Kolmogorov–Smirnov tests were used for checking the normality distributions of the data. Serum and vaginal markers (hsCRP, IGFBP-1 and MMP-8) which were not normally distributed were natural log-transformed since we wanted to apply parametric tests, which can be adjusted and are statistically more powerful compared to non-parametric tests. A one-way analysis of variance (ANOVA) test was used for analyzing parametric variables, the Kruskal–Wallis test was used for non-parametric variables, and Pearson chi-square for categorical variables. The differences in all women from early to late pregnancy were tested with ANOVA intervention groups as a fixed factor and the differences between study groups from early to late pregnancy and at late pregnancy were evaluated with one-way ANOVA. Pearson correlation was used to study the associations between markers. The markers of interest were compared between women remaining healthy and those who developed GDM with ANOVA intervention group as a fixed factor. ANOVA analyses were not adjusted with insulin since we aimed to test whether phIGFBP-1 and IGFBP-1 could be independent markers of GDM. Receiver operating characteristics (ROC) analyses were conducted to investigate the predictive value of phIGFBP-1 and IGFBP-1 and the area under the curve (AUC) was calculated. ANOVA was used to study the interaction between pre-pregnancy BMI and the intervention as well as between GDM and the intervention (BMI or GDM × intervention group interaction) on the change in serum hsCRP, phIGFBP-1, IGFBP-1, MMP-8 and vaginal MMP-8 as well as the markers measured in late pregnancy. Furthermore, to test which intervention groups were affected by a confounding factor, the independent-samples *t*-test and one-way ANOVA were used. The independent-samples *t*-test was used to study the differences in the markers between overweight and obese pregnant women in the intervention groups. One-way ANOVA Tukey’s post hoc test was used to study the impact of the intervention on the markers according to the GDM diagnosis. Results are shown as mean ± SD or (95%CI), median (IQR), percentage (%) or AUC (95%CI). *p* < 0.05 is considered statistically significant. SPSS Statistics 24.0 (IBM, Chicago, IL, USA) for Windows was used for statistical analyses.

## 3. Results

### 3.1. Clinical Characteristics

The mean age of the pregnant women was 30.6 ± 4.6 years. The majority of the tested pregnant women were overweight (60.4%), with the remainder (39.6%) being obese; somewhat more than half (54.8%) of the women were highly educated with college or university degrees, and almost half of the women (48.2%) were expecting their first child. GDM was diagnosed in 23.1% of the women in late pregnancy [26]. The pregnant women were normotensive (systolic blood pressure, 117.0 ± 10.3 mmHg; diastolic blood pressure, 76.6 ± 8.4 mmHg) and only 22% of women smoked before pregnancy.

### 3.2. The Impact of the Dietary Intervention on hsCRP, IGFBP-1 and MMP-8

The fish oil and/or probiotics intervention exerted no impact on the concentrations of serum hsCRP or serum MMP-8, phIGFBP-1 and IGFBP-1 or vaginal MMP-8 (Table 1). However, the impact of the intervention on the change in serum IGFBP-1 and MMP-8 was influenced by pre-pregnancy BMI (*p* = 0.04 and *p* = 0.03 for BMI × intervention group interaction, respectively). This was related to the differences in these markers between overweight and obese pregnant women in the probiotics + placebo and fish oil + probiotics group: in the probiotics + placebo group, MMP-8 decreased in obese pregnant women, whereas there was an increase in the overweight pregnant women (*p* = 0.02). In the fish oil + probiotics group, the concentration of IGFBP-1 increased more in obese as compared to overweight (*p* = 0.008) pregnant women, whereas the level of MMP-8 decreased more in obese compared to overweight (*p* = 0.03) women during pregnancy (Table 2).

However, the GDM × intervention group interaction was statistically significant with respect to serum phIGFBP-1 measured in late pregnancy (*p* = 0.02); this was attributable to the statistically significantly higher concentration in the fish oil + probiotics group (mean 1248.10 ± SD 391.48 ng/mL) as compared to the fish oil + placebo group (mean 854.41 ± SD 392.33 ng/mL) in women who developed GDM in late pregnancy (*p* = 0.03).

When the groups consuming fish oil (fish oil + placebo and fish oil + probiotics) were compared to groups not consuming fish oil (probiotics + placebo and placebo + placebo), serum hsCRP, phIGFBP-1, IGFBP-1 and MMP-8 and vaginal MMP-8 in early, late or during pregnancy were not different between the groups. The same result was also seen when the groups consuming probiotics (probiotics + placebo and fish oil + probiotics) and not (fish oil + placebo and placebo + placebo) were compared (results not shown).

### 3.3. HsCRP, IGFBP-1 and MMP-8 as Predictors of GDM

Serum levels of phIGFBP-1 and IGFBP-1 were related to the onset of GDM. Women who developed GDM in later pregnancy had lower concentrations of phIGFBP-1 (*p* = 0.005) and IGFBP-1 (*p* = 0.042) in early pregnancy than those women who remained healthy (Table 3). The predictive values of the markers for GDM were as follows: an AUC value for serum phIGFBP-1 of 0.596 (95% CI 0.53 to 0.67); for IGFBP-1, it was 0.572 (95%CI 0.50 to 0.64), as evaluated with ROC analysis. The serum hsCRP or vaginal MMP-8 at early pregnancy were not related to the onset of GDM (Table 3).

However, when examining the changes in the levels of hsCRP, phIGFBP-1, IGFBP-1 or MMP-8 during pregnancy according to the diagnosis of GDM, the concentration of hsCRP was reduced from early to late pregnancy to a greater extent in the women who developed GDM (natural log-transformed mean change −0.47 (95%CI −0.62 to −0.33) mg/L) than in the women who remained healthy (natural log-transformed mean change −0.25 (95%CI −0.33 to −0.17) mg/L, *p* = 0.01).

Additionally, the concentration of serum phIGFBP-1 analyzed in late pregnancy was statistically significantly lower in the women with GDM (mean 1103.72 ± SD 435.50 ng/mL) as compared to women without GDM (mean 1221.34 ± SD 449.60 ng/mL, *p* = 0.046) in late pregnancy. The levels of hsCRP, IGFBP-1 or MMP-8 did not differ between women with GDM and those without in late pregnancy (results not shown).

### 3.4. Evolution of Low-Grade Inflammation, IGFBP-1 and MMP-8 over Pregnancy

As presented in Table 2, the low-grade inflammation measured as serum hsCRP (natural log-transformed mean change −0.31 (95%CI −0.38 to −0.24) ng/mL) decreased from early to late pregnancy (Figure S1). The concentration of serum MMP-8 (natural log-transformed mean change −0.11 (95%CI −0.19 to −0.03) ng/mL) decreased whereas serum phIGFBP-1 (mean change 469.57 (95%CI 430.62 to 508.53) ng/mL) and IGFBP-1 (natural log-transformed mean change 0.33 (95%CI 0.27 to 0.38) ng/mL) increased from early to late pregnancy. In contrast, no change in the vaginal MMP-8 level (natural log-transformed mean change 0.21 (95%CI −0.09 to 0.50) ng/mL) was detected during the pregnancy.

Vaginal phIGFBP-1 was detected in only 27 of the analyzed samples (*n* = 115) in early pregnancy and in 42 samples (out of 122 samples) at late pregnancy, while IGFBP-1 was measurable in only 14 samples in early (out of 115 samples) and in 16 samples (out of 122 samples) in late pregnancy, and therefore further statistical analyses were conducted only for MMP-8.

The serum markers did not correlate with vaginal MMP-8 either in early or late pregnancy (data not shown). On the other hand, weak correlations between serum hsCRP and phIGFBP-1, IGFBP-1 and MMP-8 were detected. The level of hsCRP measured in early pregnancy correlated inversely with the serum phIGFBP-1 concentration in early and late pregnancy (*r* = −0.131, *p* = 0.007, *r* = −0.206, *p* < 0.001, respectively) and positively with serum IGFBP-1 (*r* = 0.121, *p* = 0.02) and inversely with serum MMP-8 (*r* = −0.189, *p* < 0.001) levels in late pregnancy. In late pregnancy, the hsCRP level correlated with the level of serum MMP-8 (*r* = 0.137, *p* = 0.01) and with the change in serum MMP-8 occurring during pregnancy (*r* = 0.118, *p* = 0.03). The change in the hsCRP concentration correlated with the serum IGFBP-1 level measured in late pregnancy (*r* = 0.142, *p* = 0.008).

## 4. Discussion

We observed that serum phIGFBP-1 and IGFBP-1 were related to the onset of GDM. The serum levels of both these markers were lower in early pregnancy in women who later developed GDM as compared to those who remained healthy. Moreover, phIGFBP-1 was also lower in late pregnancy in women with established GDM compared to healthy women. Instead, the administration of fish oil and/or probiotics from early pregnancy onwards did not modify per se the change occurring during the pregnancy of serum low-grade inflammation or the other measured serum markers. Interestingly, the effect of intervention on phIGFBP-1, IGFBP-1 and MMP-8 showed an interaction with the maternal BMI and on whether the mother would develop GDM. As GDM is detrimental to the health of the mother and child, means for early identification of women at risk of GDM, such as measuring serum levels of phIGFBP-1 and IGFBP-1, could be useful for reducing the possible health risks due to this disorder.

In contrast to our hypothesis and previous positive findings for the immunomodulatory effects of fish oil [38,39,40,41] and probiotics [29,30,42] which have been studied separately in non-pregnant and pregnant adults, the dietary intervention did not influence the level of low-grade inflammation or phIGFBP-1, IGFBP-1 or MMP-8 in the overweight and obese pregnant women in this unique setting of studying either separately or a combination of both fish oil and probiotics. However, the impact of the intervention on these metabolic markers analyzed was influenced by BMI and GDM, i.e., when compared to overweight women, serum MMP-8 decreased more in obese women belonging to the probiotics + placebo and fish oil + probiotics groups and IGFBP-1 increased more in obese women in the fish oil + probiotics groups. In women with GDM, late pregnancy serum levels of phIGFBP-1 were higher in the fish oil + probiotics group when compared to the fish oil + placebo group. It seems that the combination of fish oil and probiotics may be more effective in the regulation of IGFBP and MMP systems as compared to either fish oil or probiotics alone if one takes into account the BMI and GDM status of the women. Additionally, this could support the concept that these food supplements may be much more efficacious in diseased populations (in this case, an obese population) rather than in healthy, non-obese populations, where there may be nothing to improve. The results may be interpreted so that fish oil and probiotic co-supplementation may have an beneficial effect on IGFBP and MMP metabolism in pregnant women with high BMI or dysregulated glucose metabolism; there is a need for further studies to verify the effects of a combination of fish oil and probiotic supplementation.

In contrast to our results, fish oil has been shown to reduce hsCRP in a small group of pregnant women (*n* = 25) consuming a similar dose of EPA and DHA, about 2 g per day, from week 10 to 16 to term [15]. Furthermore, in two studies conducted in Iranian women with GDM, a low dose of *n*-3 LC-PUFA (180 mg EPA and 120 mg DHA per day) reduced the serum hsCRP level [13,43]. Previous studies with probiotics, which were mainly conducted in Asian populations, while ours investigated a European population, resulted in a significant decrease in the hsCRP level after consumption of Lactobacillaceae and Bifidobacteriaceae [11,44,45], which may be due to the different dietary behavior but also genetic background. In an overweight and obese adult population, the supplementation of *Bifidobacterium animalis* ssp. *lactis* 420 was shown to induce a tendency towards a reduction in hsCRP [46]. Although our study was initiated in early pregnancy and involved a larger population than those previous reports, we did not detect any statistically significant effect; thus, it is noteworthy that the timing and duration of the intervention and also probiotic strains or their combination may have an effect on the outcome, as well as the study population in whom the intervention is conducted. Interestingly, in a recent meta-analysis of randomized clinical trials, serum hsCRP decreased significantly in healthy adults as well as in adults with a wide range of health conditions, except for patients with metabolic diseases and those with allergy and autoimmune diseases, after probiotic supplementation as compared to placebo [47].

In our study, serum hsCRP measured in early pregnancy did not predict the onset of GDM in later pregnancy, which is in line with the report of Naser et al. [48]. In contrast, in another previous study, hsCRP levels were shown to be higher in women who subsequently develop GDM [49]. Insulin has been suggested to control the transcription of acute-phase protein genes [50] and thus Chan et al. [17] suggested that decreased insulin sensitivity could increase the expression of hsCRP, which is likely to be the case in women with GDM. Instead, we detected a greater decrease in serum hsCRP during pregnancy in those women who developed GDM as compared to those women who remained healthy, suggesting that pregnancy-induced changes in hsCRP are more established in women developing GDM. Moreover, it has been shown that in both obese pregnant women and those with GDM, the inflammatory profiles are altered, not necessarily only increased but also decreased, as compared to healthy normal weight women during pregnancy [51].

In terms of GDM and IGFPB-1, our results are in line with those of Qui et al. [52], who showed that IGFBP-1 levels correlated inversely with the risk of developing GDM. In another study, the extent of phosphorylation of cord blood phIGFBP-1 was related to the levels detected in GDM and non-GDM women: highly phosphorylated IGFBP-1 levels decreased in women with GDM, but not those of the lesser phosphorylated IGFBP-1 as compared to healthy women in late pregnancy [53]. In our study, the phosphorylated form of IGFBP-1 was lower in women with GDM as compared to their healthy counterparts at late pregnancy and was a more effective predictor of GDM than the non-phosphorylated form, supporting the previous findings of phIGFBP in GDM. Considering the non-phosphorylated IGFBP-1, Liao and co-workers [54] found lower levels of IGFBP-1 concentrations at week 20 of gestation in women with GDM in comparison to controls. However, in another study, there were higher levels of IGFBP-1 in women with GDM than in their healthy counterparts, but this study was conducted at 24–28 weeks of gestation [55].

In diabetes, including GDM, increased insulin levels may potentially lead to decreased levels of IGFBP-1. IGFBP-1 affects glucose homeostasis by regulating the glucose lowering effect of IGF-1. In experimental studies, it has been shown that exogenous administration of IGF-1 results in enhanced insulin sensitivity and glucose uptake [9]. IGF-1 shares structural components and effects with insulin and the bioavailability of IGF-1 is regulated by insulin. Additionally, the production of IGFBP-1 is regulated by insulin [2]. In our study, IGFBP-1 as well as phIGFBP-1 were lower in early pregnancy in the women who developed GDM in later pregnancy as compared to women who did not. We suggest that the lower levels of early pregnancy may be related to the development of GDM in later pregnancy, as we showed that low levels in early pregnancy remained low in women who developed GDM in later pregnancy. It is possible that the phosphorylated form is responsible for these effects, since the extent of phosphorylation of IGFBP-1 has an impact on the affinity of IGF-1 for IGFBP-1 [53], thus further regulating the bioavailability of IGF-1. The mechanism by which MMP-8 is related to obesity and insulin resistance is not completely clarified. In this regard, MMP-8 can proteolytically modify insulin-receptor leading potentially to development of insulin resistance [10].

Mechanisms between IGFBP-1, phIGFBP-1, and MMP-8 and probiotics and/or fish oil are not known in detail. Probiotics may affect the protein synthesis of IGFBPs [22], while n-3 LC-PUFA stimulate MMP production via prostaglandin production [56]. Other possible mechanisms may relate to the short chain fatty acid production of probiotics and anti-inflammatory properties of fish oil as suggested by Yan et al. [57] and by Gholamhosseini et al. [20], respectively. To our knowledge, not much is known about the dietary regulators of IGFBP-1 and MMP-8. One study showed that carbohydrates, plant protein and milk protein were directly associated with IGFBP-1 [58], and protein restriction has been shown to decrease the levels of IGFBP-1 [59]. Regarding MMP-8, the studies investigating the impact of diet on MMP-8 are lacking. Previously, we investigated phIGFBP-1, IGFBP-1 and MMP-8 in smaller number of pregnant women (*n* = 100) and we found that polyunsaturated fatty acids correlated directly with IGFBP-1 and potassium and pyridoxine inversely with MMP-8 [7].

We showed that serum hsCRP and MMP-8 decreased while IGFBP-1 and phIGFBP-1 increased during pregnancy, regardless of the intervention or GDM status. Our findings with respect to the changes of IGFBP-1 and hsCRP during pregnancy are in agreement with the results of previous studies [60,61,62,63] but some discrepancies exist regarding hsCRP, e.g., a study conducted in non-obese pregnant women with GDM detected an elevation in hsCRP along with the gestational age [64]. The MMP-8 level declined, which is in contrast to previous studies in which increased MMP-8 levels were related to adverse pregnancy outcomes including preterm delivery [65] or high amniotic fluid MMP-8 in intra-amniotic inflammation [5]. However, the levels of MMP-8 as well as those of phIGFBP-1 and IGFBP-1 correlated with hsCRP in our study, and previously the levels of IGF-1 and IGFBPs, such as IGFBP-4, have been shown to associate with those of CRP and IL-6 [66]. Thus, our study and previous reports indicate that the IGF-system may be linked to low-grade inflammation.

In terms of vaginal samples, we did not detect IGFBP but did find evidence of MMP-8 in both the early and the late pregnancy samples. We did not find any difference between the first and the second measurement of MMP-8 during pregnancy, nor did Rahkonen et al. [67] although they detected an association between an increased MMP-8 level and bacterial vaginosis and leukocytosis in the first and the second trimester suggesting a link between MMP-8 and elevated levels of inflammation. We measured low-grade inflammation as reflected by hsCRP but did not find any association between vaginal MMP-8 and hsCRP levels. These results indicate that the inflammatory state during gestation is not stable but instead tends to vary, depending on many factors, such as BMI [60].

In our study, we did not find differences between the intervention groups in the amount of bleeding (>1000 mL); this result was reported in Pellonperä et al. [26]. Moreover, other adverse effects were very minimal e.g., headache or gastrointestinal symptoms. Thus, the intervention was safe and well tolerated. These findings are in line with previous reports [68] and European Food Safety Authority evaluation [69], i.e., it is safe to consume fish oil during pregnancy. The strengths of our study lie in its unique four group factorial design with two active dietary ingredients, fish oil and probiotics, and in a well-characterized study population, as well as an evaluation of the markers in both the serum and vagina. As far as we are aware, this is the first clinical trial investigating the synergistic benefits of fish oil and probiotics in pregnant women. However, this is a secondary analysis of the main trial. Yet, previous studies investigating the impact of the dietary intervention on low-grade inflammation included lower number of patients (ranging from 40 to 72) than ours and still found significant results [11,13,14,15,42,43,44,45]. Furthermore, unlike the previous studies, we took into consideration the overweight and obesity status. We aimed to study an overweight and obese group of pregnant women at risk for gestational diabetes and it remains for further studies to demonstrate if similar findings are seen in normal weight pregnant women, which is one limitation of our study. Other limitations could be the small number of vaginal samples, the fact that secondary outcomes of the main trial were investigated, and that we only studied hsCRP as a marker for inflammation. We could not detect the levels of IGFBP-1 and phIGFBP-1 in the vaginal samples which could be due to the sample number or very low levels in this group with a low prevalence of preterm deliveries, but this finding needs to be confirmed.

## 5. Conclusions

The extent of low-grade inflammation, as determined by hsCRP, and the levels of the metabolic markers, IGFBP-1, phIGFBP-1 and MMP-8, were altered during pregnancy, indicating changes in inflammatory and metabolic status in overweight and obese women throughout gestation. This change was not influenced by our fish oil and/or probiotic intervention, but it was noted that there was an interaction between how the intervention altered the metabolic markers depending on the women’s obesity status and whether or not they developed GDM. Our observations call for further studies in order to clarify the potential benefits of this kind of intervention in pregnant women, particularly those at an increased risk for health complications. The serum concentrations of IGFBP-1 and phIGFBP-1 were lower in early pregnancy in those women who subsequently developed GDM in later pregnancy, suggesting their potential as early markers for GDM. The result of our study together with previous reports indicate that IGFBPs may have a role in the development of GDM via the regulation of the glucose and insulin metabolism. At the same time, supplementing the diet of pregnant women with fish oil and/or probiotics supplementation needs to be investigated further, since we showed that serum levels of IGFBP and MMP vary depending not only on the BMI and GDM status but also on whether the pregnant woman received a supplementation with fish oil and/or probiotics.

## 6. Patents

T.S. and J.J. are inventors of diagnostic patents 127416 and US 2017/0023671A1 (MMP-8 serum test).

## Figures and Tables

**Table 1:** The concentrations of early and late pregnancy serum high sensitivity C-reactive protein (hsCRP), matrix metalloproteinase 8 (MMP-8), phosphorylated insulin-like growth factor binding-protein 1 (phIGFBP-1), IGFBP-1 and vaginal MMP-8 in the different intervention groups and in all of the women. Data are expressed as mean ± SD and 95% Confidence Intervals for mean change (95%CI).

	Fish Oil + Placebo	Probiotics + Placebo	Fish Oil + Probiotics	Placebo + Placebo	All		
Serum Markers						Subjects (*n*)	*p* Value ^a^
hsCRP (mg/L) ^b^							
Early pregnancy	1.68 ± 0.76	1.63 ± 0.79	1.58 ± 0.74	1.57 ± 0.80	1.61 ± 0.77	108/109/108/109/434	
Late pregnancy	1.39 ± 0.85	1.32 ± 0.80	1.29 ± 0.78	1.26 ± 0.74	1.32 ± 0.79	93/94/90/92/369	0.72
Mean change	−0.29	−0.33	−0.32	−0.30	−0.31 ^c^	92/94/90/91/367	0.97
95%CI	−0.43–(−0.15)	−0.46–(−0.20)	−0.45–(−0.18)	−0.44–(−0.16)	−0.38–(−0.24) ^c^		
MMP-8 (ng/mL) ^b^							
Early pregnancy	3.04 ± 0.66	2.90 ± 0.64	2.89 ± 0.60	2.87 ± 0.58	2.93 ± 0.62	107/106/106/108/427	
Late pregnancy	2.87 ± 0.73	2.79 ± 0.64	2.75 ± 0.71	2.83 ± 0.70	2.81 ± 0.70	89/89/90/87/355	0.68
Mean change	−0.15	−0.11	−0.13	−0.06	−0.11 ^c^	87/86/89/86/348	0.89
95%CI	−0.30–0.10	−0.28–0.06	−0.27–0.02	−0.22–0.09	−0.19–(−0.03) ^c^		
phIGFBP-1 (ng/mL)							
Early pregnancy	708.53 ± 343.32	701.59 ± 340.79	702.04 ± 309.79	696.04 ± 355.21	702.04 ± 336.64	107/107/106/108/428	
Late pregnancy	1160.02 ± 509.38	1236.36 ± 489.00	1179.84 ± 422.35	1153.10 ± 380.46	1182.34 ± 452.68	89/88/90/87/354	0.61
Mean change	452.97	490.88	466.85	467.59	469.57 ^c^	87/86/89/86/348	0.93
95%CI	377.67–528.26	404.56–577.21	379.56–554.15	404.64–530.54	430.62–508.53 ^c^		
IGFBP-1 (ng/mL) ^b^							
Early pregnancy	3.85 ± 0.74	3.84 ± 0.78	3.90 ± 0.73	3.90 ± 0.70	3.87 ± 0.74	107/106/105/108/427	
Late pregnancy	4.16 ± 0.63	4.26 ± 0.56	4.23 ± 0.56	4.21 ± 0.60	4.21 ± 0.58	89/88/90/86/353	0.72
Mean change	0.28	0.35	0.33	0.34	0.33 ^c^	87/85/89/85/348	0.83
95%CI	0.17–0.39	0.24–0.46	0.21–0.44	0.22–0.46	0.27–0.38 ^c^		
Vaginal markers							
MMP-8 (ng/mL) ^b^							
Early pregnancy	3.45 ± 1.62	3.22 ± 1.46	3.06 ± 1.95	3.46 ± 1.59	3.29 ± 1.65	28/29/29/28/115	
Late pregnancy	3.44 ± 1.34	3.96 ± 1.48	3.33 ± 1.67	3.64 ± 1.21	3.61 ± 1.44	35/35/27/27/124	0.31
Mean change	0.04	0.55	0.26	−0.04	0.21 ^c^	25/24/19/17/85	0.48
95%CI	−0.55−0.63	0.16–0.93	−0.41–0.98	−0.88–0.79	−0.09–0.50 ^c^		

^a^ Test between the intervention groups: one-way ANOVA. ^b^ Natural log-transformed variables. ^c^ Adjusted for intervention.

**Table 2:** The change in the serum levels of matrix metalloproteinase 8 (MMP-8) and insulin-like growth factor binding-protein 1 (IGFBP-1) (mean change (95%CI)) from early to late pregnancy in overweight and obese pregnant women subdivided according to the four intervention groups.

	Overweight Pregnant Women	Obese Pregnant Women	*n*	*p* Value ^a^
Serum MMP-8 (ng/mL) ^b^				
Fish oil + Placebo	−0.17 (−0.38–0.05)	−0.12 (−0.35–0.12)	47/40	0.74
Probiotics + Placebo	0.33 (−0.17–0.23)	−0.40 (−0.70–(-0.11))	58/28	0.02
Fish oil + Probiotics	−0.005 (−0.17–0.16)	−0.33 (−0.62–(-0.04))	56/33	0.03
Placebo + Placebo	−0.12 (−0.32–0.08)	0.02 (−0.24–0.28)	51/35	0.40
Serum IGFBP-1 (ng/mL) ^b^				
Fish oil + Placebo	0.32 (0.18–0.47)	0.23 (0.05–0.42)	47/40	0.44
Probiotics + Placebo	0.32 (0.18–0.45)	0.42 (0.23–0.62)	57/28	0.36
Fish oil + Probiotics	0.20 (0.07–0.33)	0.54 (0.33–0.76)	56/33	0.008
Placebo + Placebo	0.35 (0.20–0.51)	0.32 (0.14–0.51)	51/34	0.81

^a^ Test between overweight and obese pregnant women: independent-samples *t*-test. ^b^ Natural log-transformed variables.

**Table 3:** The concentrations of serum matrix metalloproteinase 8 (MMP-8), phosphorylated insulin-like growth factor binding-protein 1 (phIGFBP-1) and IGFBP-1 and vaginal MMP-8 (mean ± SD) measured in early pregnancy in those women remaining healthy and in those who developed gestational diabetes mellitus (GDM) later in pregnancy.

	Women Remaining Healthy		Women Who Developed GDM		
Serum Markers		*n*		*n*	*p* Value ^a,c^
hsCRP (mg/L) ^b^	1.59 ± 0.79	276	1.71 ± 0.61	83	0.22
phIGFBP-1 (ng/mL)	753.24 ± 335.11	271	635.85 ± 315.29	82	0.005
IGFBP-1 (ng/mL) ^b^	3.96 ± 0.69	269	3.78 ± 0.72	82	0.042
MMP-8 (ng/mL) ^b^	2.92 ± 0.62	270	2.89 ± 0.61	82	0.77
Vaginal markers					
MMP-8 (ng/mL) ^b^	3.42 ± 1.53	69	2.97 ± 1.52	29	0.19

^a^ Test between women remaining healthy and women who developed GDM: ANOVA. ^b^ Natural log-transformed variables. ^c^ Adjusted for intervention.

## Data Availability

Data is contained within the article or supplementary material The data presented in this study are available in [insert article or supplementary material here].

## Supplementary Materials

The following are available online at https://www.mdpi.com/2218-273X/11/1/5/s1, Figure S1: a, b, c, d and e. The concentrations of serum (a) hsCRP, (b) MMP-8, (c) phIGFBP-1, (d) IGFBP-1 and (e) vaginal MMP-8 (mean ± SD) in early and late pregnancy. ANOVA, * *p* < 0.05.
