# Supplementary material for: The Impacts of Fish Oil and/or Probiotic Intervention on Low-Grade Inflammation, IGFBP-1 and MMP-8 in Pregnancy: A Randomized, Placebo-Controlled, Double-Blind Clinical Trial
Source: Biomolecules. 2020 Dec 22;11(1):5. doi: 10.3390/biom11010005 (PMC7822218; doi:10.3390/biom11010005)
Supplement: Supplementary file 1 [file biomolecules-11-00005-s001.pdf]

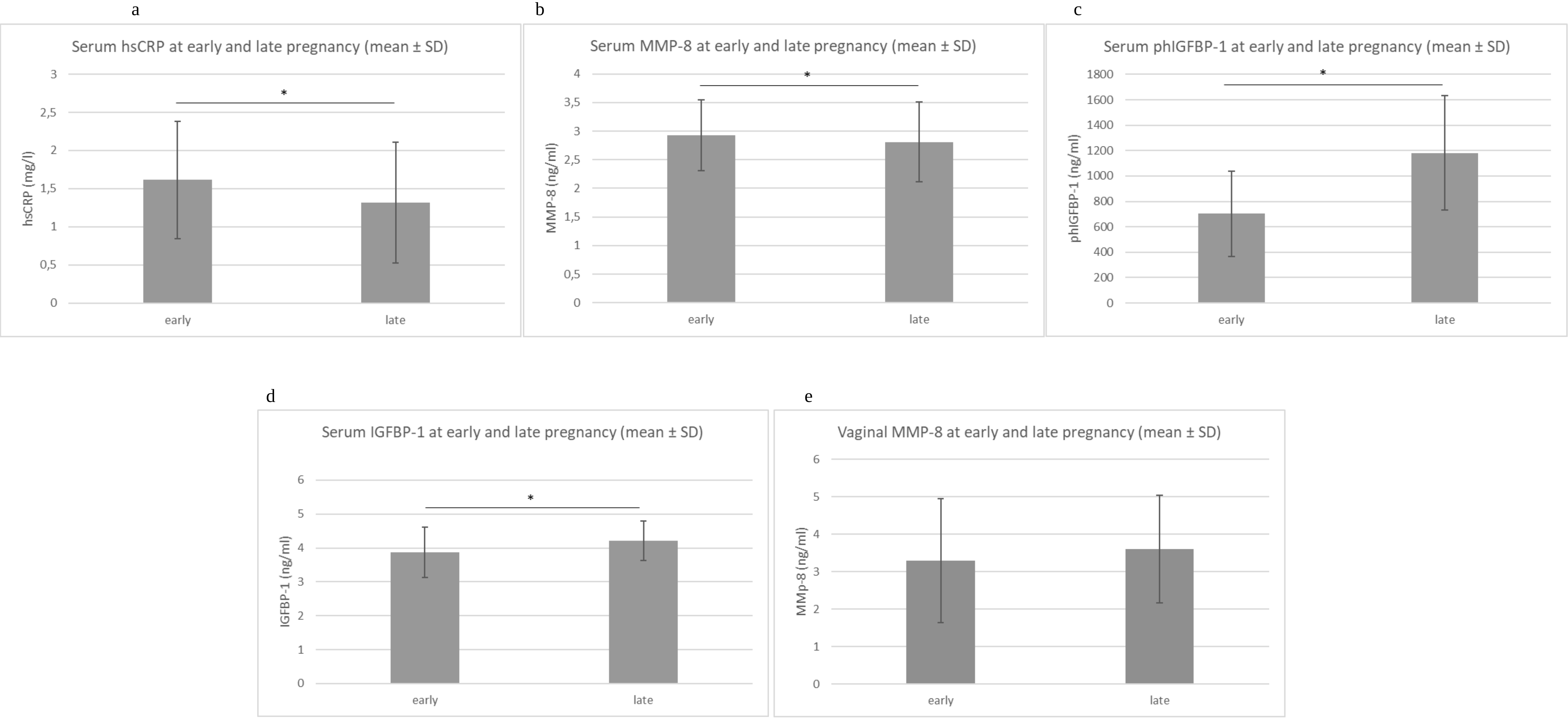

Figure S1. a, b, c, d and e. The concentrations of serum a) high sensitivity C-reactive protein (hsCRP), b) matrix metalloproteinase 8 (MMP-8), c) phosphorylated insulin-like growth factor binding-protein 1 (phIGFBP-1), d) IGFBP-1 and e) vaginal MMP-8 (mean ± SD) in early and late pregnancy. ANOVA, **p* <0.05.
